# Detecting inappropriate total duration of antimicrobial therapy using semi-automated surveillance

**DOI:** 10.1186/s13756-022-01147-2

**Published:** 2022-08-29

**Authors:** Annemieke K. van den Broek, Jara R. de la Court, Thomas Groot, Reinier M. van Hest, Caroline E. Visser, Kim C. E. Sigaloff, Rogier P. Schade, Jan M. Prins

**Affiliations:** 1 Division of Infectious Diseases, Department of Internal Medicine, Amsterdam UMC, University of Amsterdam, Vrije Universiteit Amsterdam, Amsterdam, The Netherlands; 2 Department of Medical Microbiology and Infection Prevention, Amsterdam UMC, University of Amsterdam, Vrije Universiteit Amsterdam, Amsterdam, The Netherlands; 3 Division of Clinical Pharmacology, Department of Hospital Pharmacy, Amsterdam UMC, University of Amsterdam, Amsterdam, The Netherlands

## Abstract

**Objectives:**

Evaluation of the appropriateness of the duration of antimicrobial treatment is a cornerstone of antibiotic stewardship programs, but it is time-consuming. Furthermore, it is often restricted to antibiotics prescribed during hospital admission. This study aimed to determine whether mandatory prescription-indication registration at the moment of prescribing antibiotics enables reliable automated assessment of the duration of antibiotic therapy, including post-discharge duration, limiting the need for manual chart review to data validation.

**Methods:**

Antibiotic prescription and admission data, from 1-6-2020 to 31-12-2021, were electronically extracted from the Electronic Medical Record of two hospitals using mandatory indication registration. All consecutively prescribed antibiotics of adult patients who received empiric therapy in the first 24 h of admission were merged to calculate the total length of therapy (LOT) per patient, broken down per registered indication. Endpoints were the accuracy of the data, evaluated by comparing the extracted LOT and registered indication with the clinical notes in 400 randomly selected records, and guideline adherence of treatment duration. Data were analysed using a reproducible syntax, allowing semi-automated surveillance.

**Results:**

A total of 3,466 antibiotic courses were analysed. LOT was accurately retrieved in 96% of the 400 evaluated antibiotic courses. The registered indication did not match chart review in 17% of antibiotic courses, of which only half affected the assessment of guideline adherence. On average, in 44% of patients treatment was continued post-discharge, accounting for 60% (± 19%) of their total LOT. Guideline adherence ranged from 26 to 75% across indications.

**Conclusions:**

Mandatory prescription-indication registration data can be used to reliably assess total treatment course duration, including post-discharge antibiotic duration, allowing semi-automated surveillance.

**Supplementary Information:**

The online version contains supplementary material available at 10.1186/s13756-022-01147-2.

## Background

Antimicrobial Stewardship Programs (ASP) aim to reduce antimicrobial resistance and its associated morbidity, mortality and healthcare costs [1]. One of the elements of ASPs is to promote prescription of the shortest effective duration of antibiotic therapy [2]. Antibiotic courses that are longer than necessary increase the selective pressure on bacterial flora [3]. and each additional day of antibiotic use may increase the risk of patient harm [4]. It is therefore important to monitor the appropriateness of antibiotic course durations, in order to identify target-areas for improvement. The few studies evaluating treatment duration (including post-discharge antibiotic use) in general described the average quantity of antibiotic use or length of therapy, irrespective of indication and without evaluating guideline adherence [5, 6].

An in-depth audit assessing the appropriateness of choice and duration of antibiotic therapy for each individual antibiotic course until recently required manual data collection from the (electronic) medical record (EMR), which is time-consuming [7, 8]. As a result, the number of antibiotic prescriptions that could be evaluated was limited by available personnel and resources [7, 8] Recently, the use of EMR to facilitate automated ASP audits has grown in relevance, as it was demonstrated that this can provide data that enable efficient measurement of the appropriateness of antibiotic use [9, 10]. As a result, a considerable amount of manual chart review can be disregarded, reducing labour intensity*.* Utilizing indication selected at time of order entry also enable stewardship programs perform targeted real-time interventions, as many other methods (like using ICD-10 s) can only occur post-discharge. Dyer and colleagues showed that it is feasible to extract from the EMR the total duration of antibiotic use prescribed to inpatients per registered ICD-10 diagnosis, including post-discharge prescriptions [6]. It is important to consider these post-discharge prescriptions, as they may take up to 40% of the total antibiotic course [5]. Unfortunately, they did not evaluate the appropriateness of treatment duration.

In a previous study we showed that it is feasible to link antibiotic indications to antibiotic prescriptions by mandatory indication registration, which enables systematic evaluation and benchmarking of the appropriateness of the empiric antimicrobial choice for respiratory tract infections (RTI) and urinary tract infections (UTI) [10, 11]. The aim of the present study was to demonstrate that it is also possible to reliably determine the total duration of antibiotic treatment prescribed to hospital inpatients, including post-discharge duration, broken down per indication, using data extracted from the EMR. The secondary aim was to determine whether the extracted data can be used to evaluate guideline adherence of treatment duration.

## Methods

### Study design and setting

A retrospective observational study was performed on data obtained from the Amsterdam University Medical Centres (Amsterdam UMC), a university-affiliated tertiary care hospital with two locations: AMC and VUmc. Both locations use EPIC software as their electronic medical record and prescribing software. In 2019, a standardized prescription-format (Additional file 1), was implemented in the EMR, as described in our previous study [10]. This prescription-format requires physicians to select the indication for the prescription from a predefined list whenever they prescribe an antimicrobial agent for hospital inpatients. Approval from the Institutional review boards was not required for this study because we used retrospective, pseudonymised data for quality optimization purposes. Procedures were in accordance with the General Data Protection Regulation [12].

**Table 1:** Accuracy of extracted duration and selected indications

	Duration of therapy (inaccurate duration/number of screened records)	Indication of therapy (inaccurate selections/number of screened records)
Error rate location AMC (%)	10/200 = 5.0% 6 prescriptions not electronically prescribed (all were OPAT prescriptions) 1 prescription of which start fell before inclusion paeriod and therefore not included in the dataset 1 missing non-J01 antibiotic (oral metronidazole) 2 prescriptions were not terminated after discharge or death *18 transfers to other hospital leading to missing data*	31/200 = 15.5% Incorrectly registered indications affecting duration of therapy: 12/31 = 38.7%^a^
Error rate location VUMC (%)	8/200 = 4.0% 8 prescriptions not electronically prescribed (of which 5 were OPAT prescriptions) *14 transfers to other hospital leading to missing data*	37/200 = 18.5% Incorrectly registered indications affecting duration of therapy: 23/37 = 62.0%^c^
Total	18/400 = 4.5%	68/400 = 17.0% Incorrectly registered indications affecting duration of therapy: 35/400 = 8.8%

*OPAT* Outpatient parenteral antimicrobial therapy

### Data collection and definitions

Data covering the period from 1-6-2020 to 31-12-2021 was extracted from the EMR. The extracted data contained all antibiotic prescription orders for systemic use belonging to Anatomical Therapeutic Chemical (ATC) class J01, the prescription-linked registered indication on tract level (anatomical location of the disease) and a further specification in case of respiratory tract infections (RTI) and urinary tract infections (UTI), the duration of hospital admission (time and date of admission and discharge), the duration of therapy (start and stop date, including the post-discharge period), and the specialty of the prescribing physician.

**Fig. 1:**
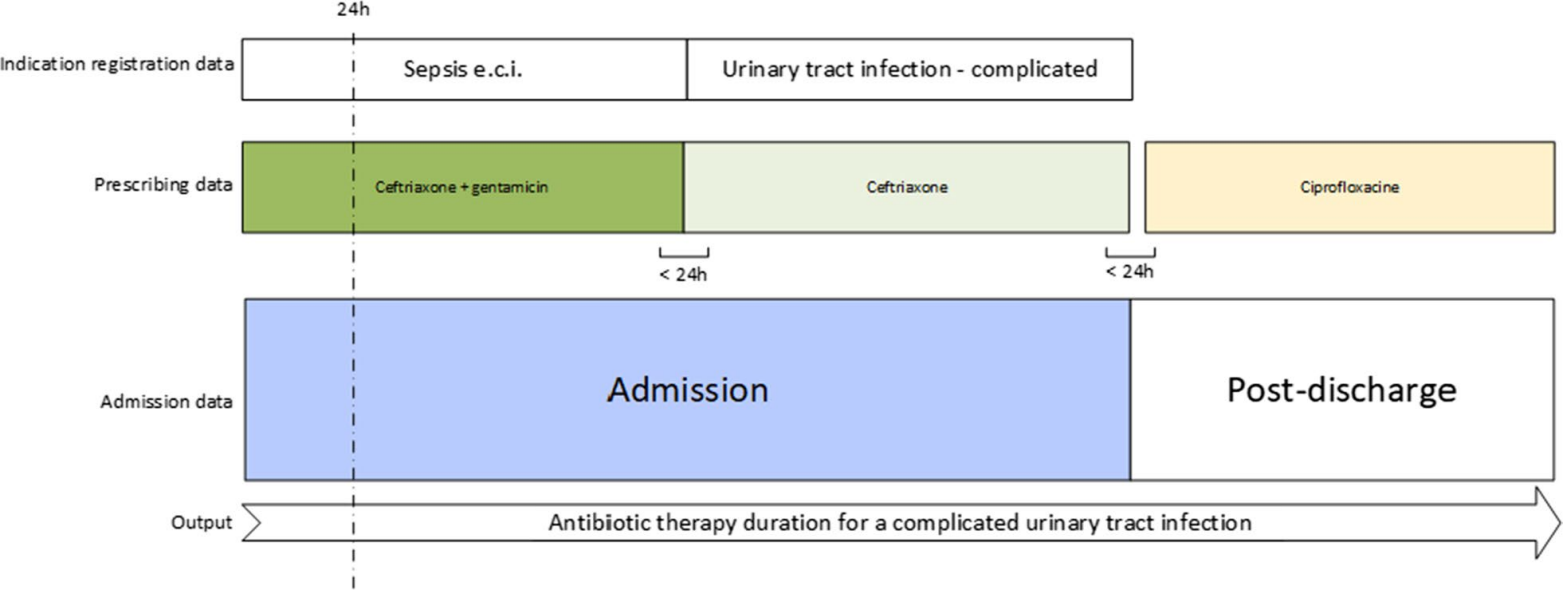
Defining the total antibiotic therapy duration for the definitive registered indication

For the purpose of this study, we identified all antibiotic prescription in patients admitted to any general ward who were prescribed empirical antibiotic treatment. Hospitalized patients were defined as patients that were admitted for at least 12 h. Empirical antibiotic treatment was defined as the prescription of systemic antibiotics at time point 24 h of hospitalization, or at the time of discharge in patients who were hospitalized for 12–24 h. These empirical inpatient prescription orders with a registered indication were merged with consecutively prescribed antibiotics during hospital admission and post-discharge, to define the total antibiotic treatment course per patient. The last registered indication during the admission was considered as the definitive indication for the full treatment course (Fig. 1), because the treatment indication can initially change as new results become available. Antibiotics that were prescribed simultaneously for the same specified indication were considered as antibiotic combination therapy. We regarded outpatient antibiotic prescriptions that were initiated within a maximum interval of 24 h after the final dosage of the last inpatient antibiotic prescription as part of the antibiotic course for the definitive indication.

In case patients received non-consecutively antibiotic treatment during admission or within 30 days of readmission, only the antibiotic course for the initial episode was included. Prescriptions that were prescribed for longer than 21 days or for indications that in general require a treatment duration longer than 21 days were excluded, as the optimal treatment duration in these cases is usually guided by the characteristics of that particular patient. In addition, during initial validation of the extracted datasets, we observed that a prolonged duration was often caused by prescribed prophylaxis after discharge. Antibiotics that were linked to the indication prophylaxis were excluded. We also excluded paediatric patients (age < 18 years) and patients admitted to the Intensive Care Unit, because guideline-recommended treatment is usually not applicable in this setting, as well as patients who received antibiotics for two different indications that were registered simultaneously.

### Validation of the dataset

**Fig. 2:**
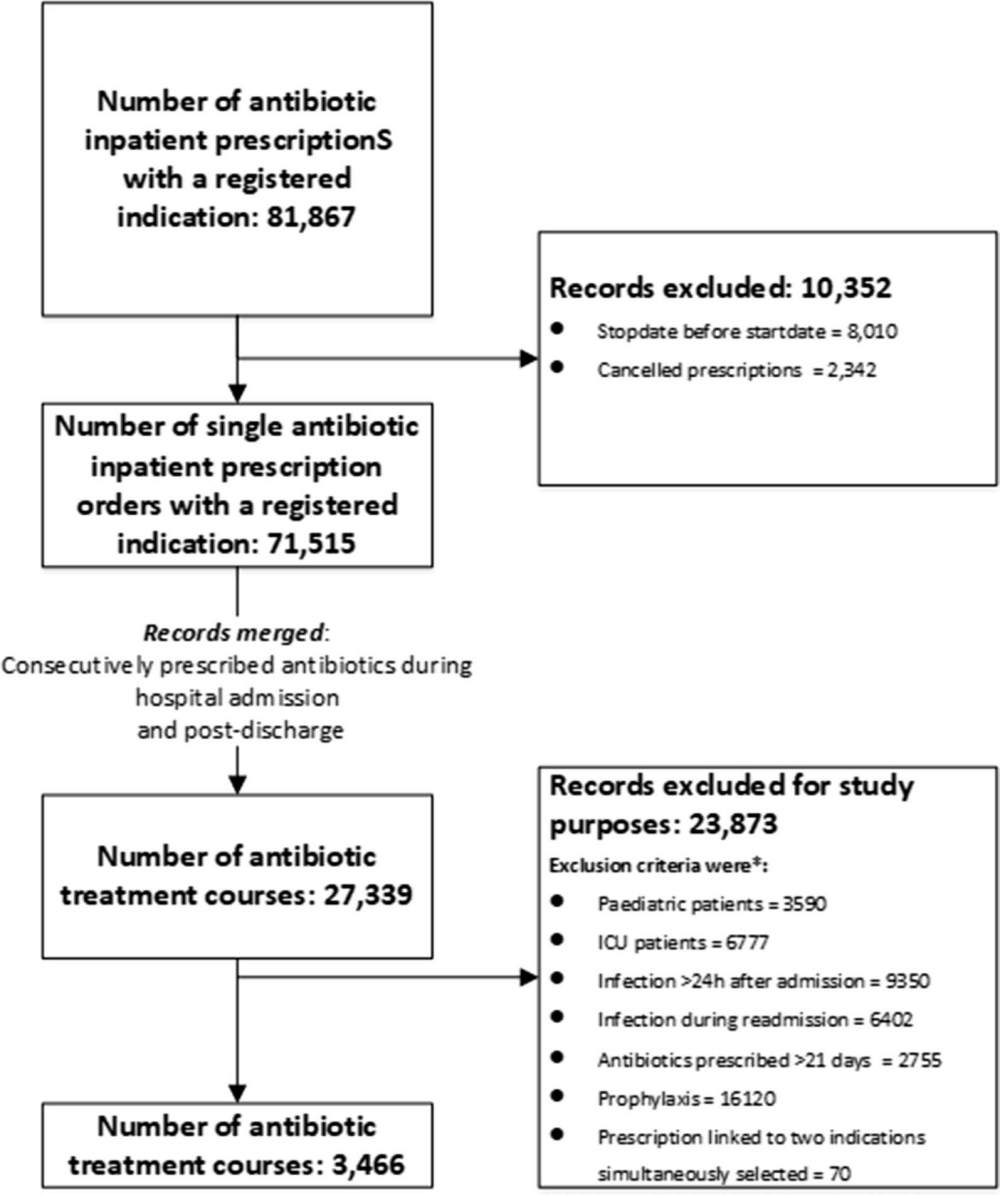
Overview of the data selection steps resulting in the final dataset. *Exclusion criteria may overlap

We first extracted all aforementioned antibiotic prescribing and hospital admission data from the EMR and selected the records meeting our inclusion criteria (Fig. 2). We inspected the correctness of all extracted data first by looking at the extracted data to investigate what data was actually extracted from the EMR and whether the extracted data provided the information that we were looking for. And if necessary, manual chart review of a number of records, to adjust the data selection until the output met all inclusion criteria. Thereafter, accuracy of the dataset was verified by manually screening a sample of 400 (200 per location) randomly selected records of patients who fulfilled the inclusion criteria. We checked whether the extracted indication and total duration of antibiotic therapy were in accordance with the indication and duration of therapy as documented in the EMR. We hereby distinguished between: inaccurately selected indications in general and inaccurately selected indications that would affect the assessment of total treatment duration, which was the case when the recommended treatment duration differed between the inaccurately selected indication and the actual indication as documented in the clinical notes.

### Study endpoints

The primary endpoint was the accuracy of the electronically obtained length of therapy (LOT). LOT was defined as the number of calendar days during which antimicrobials were consecutively prescribed for the definitive indication, including post-discharge duration, irrespective of the number of agents or doses on each calendar day. Accuracy was defined as percentage agreement between 1) the electronically obtained LOT and the manually retrieved LOT from the EMR; 2) the registered indication and the manually retrieved indication from the EMR, as for determination of guideline adherence the registered indication has to be correct. Secondary endpoint was the percentage of antibiotic treatment courses of which the LOT was according to local guidelines. The local guidelines are based on the national guidelines of the Dutch Working party on Antibiotic Policy (www.amsterdamumc.swab-id.nl). When the LOT fell within the guideline-recommended treatment duration range we considered it guideline-adherent. We added a margin of one day, as days of therapy are registered irrespective of the number of doses (e.g., administration of a single dosage in the evening adds a full day to the LOT).

### Data analysis

The median LOT and interquartile range (IQR) are presented for each registered indication. We provide total LOT. We calculated per indication the percentage of treatment courses in which part of the treatment was given post-discharge. For these courses we provided the proportion (± SD) of the total treatment duration that was given post-discharge. The proportion of guideline (non)-adherent prescriptions is expressed as percentage of the total number of antimicrobial prescriptions for that indication. All data handling and visualisation was performed using TIBCO® Spotfire®.

## Results

### Dataset characteristics and validation

The extracted data from the EMR yielded a total of 142,470 systemic antibiotic prescriptions (J01) (including post-discharge and outpatient clinic prescriptions that were not linked to an indication). Of these, 81,867 systemic antibiotic prescriptions (J01) were prescribed during hospital admission and were therefore linked to a registered indication. After general inspection of the data, including manual chart review, we excluded erroneous prescriptions, defined as prescriptions of which the start date of the antibiotic fell before the date of admission. Also, prescriptions that were labelled as cancelled were excluded, as these were not administered to patients. The remaining 71,515 prescribed systemic antibiotics (J01) represented 27,399 antibiotic courses. Applying our pre-defined in- and exclusion criteria resulted in 3,466 antibiotic courses for further analysis. A schematic overview of the selection steps and yield is presented in Fig. 2. The used syntax (pseudo-code) is available on request.

### Data validation

Of the final dataset, a random sample of 400 records was evaluated on accuracy (Table 1). Overall, in only 4.5% of patients the electronically extracted LOT—including the post-discharge treatment duration—did not match the total treatment duration as documented in the EMR. Most of these patients were discharged with outpatient antimicrobial IV treatment (OPAT). OPAT is not yet captured in the electronic prescribing system and therefore requires written prescriptions. 32/400 patients were transferred to another centre. Their total duration of antibiotic therapy was therefore unknown.

We also compared the selected indication with the diagnosis that was recorded in the patient record and found an error-rate of 17.3% (68/400). Although the tract of infection was mostly accurate, its specification was not always. Gastro-intestinal and intra-abdominal infections were commonly selected interchangeably. Furthermore, sometimes cystitis was selected instead of complicated UTI; severe community-acquired pneumonia (CAP) instead of mild-to-moderate CAP; fever of unknown cause instead of febrile neutropenia, or therapy instead of prophylaxis. Of these incorrectly registered indications, 51.5% affected the assessment of guideline-adherent LOT. This was mainly the case for the inaccurate selection of cystitis instead of complicated UTI or therapy when it actually concerned prophylaxis.

### Total duration of antibiotic therapy

The median LOT per indication is presented in Table 2. Note that the LOT is underestimated for infections that sometimes require prolonged antibiotic treatment for > 21 days, e.g., S. aureus bacteremia, as these patients were excluded from data analysis. Most antibiotic courses were prescribed for UTI and RTI. A large proportion of the treatment courses for ENT infections, complicated UTI and skin and soft tissue infections (SSTI) were continued post-discharge (i.e. 83%, 68% and 64% respectively), of which the post-discharge duration accounted for 62–63% of the total LOT.

**Table 2:** Length of treatment (LOT) per indication

Definitive diagnosis	Total (n)	Treatment courses with post-discharge treatment (n;%)	Percentage of total treatment duration given post-discharge (%;SD)*	LOT in days, (median; IQR)	Recommended course duration^**^	Guideline adherence^***^ (%)	Treatment courses too long (%)
UTI—Cystitis	420	231 (55)	63 (21)	6.5 (4.0–10.0)	3–7 days	53	31
UTI—Complicated UTI	241	164 (68)	63 (18)	12.0 (7.0–15.0)	7–14 days	60	15
UTI—CAD	28	15 (54)	65 (18)	11.0 (4.8–14.0)	7–14 days	57	14
UTI—Kidney transplant	47	28 (60)	59 (17)	11.0 (5.5–15.0)			
UTI—Other (not specified)	89	60 (67)	68 (18)	13.0 (7.0–15.0			
RTI—CAP-m	328	142 (43)	54 (20)	6.0 (5.0–8.0)	5 days	35	40
RTI—CAP-s	90	17 (19)	46 (24)	5.0 (3.0–7.0)	5 days	29	31
RTI—HAP	41	6 (15)	37 (24)	6.0 (4.0–8.0)	5–7 days	46	24
RTI—Aspiration	82	23 (28)	51 (20)	6.5 (5.0–9.0)	5 days	26	50
RTI—COPD	73	32 (44)	58 (18)	6.0 (3.0–8.0)	7 days	38	14
RTI—Abscess/empyema	23	9 (39)	55 (19)	8.0 (5.5–15.0)			
RTI—Other (not specified)	154	51 (33)	56 (17)	6.0 (3.0–8.8)			
CNS infection	51	7 (14)	70 (21)	8.0 (3.0–11.5)			
CVL infection	29	11 (38)	59 (17)	7.0 (4.0–13.0)			
ENT infection	106	88 (83)	63 (14)	10.0 (9.0–13.0)	7–14 days	75	10
Gastro-enteritis	40	14 (35)	60 (21)	5.0 (3.0–8.0)			
Gynaecological infection	165	91 (55)	68 (16)	7.0 (2.0–11.0)			
Intra-abdominal infection	464	145 (31)	52 (19)	6.0 (4.0–8.0)			
Sepsis e.c.i./unkown	270	53 (20)	56 (20)	4.0 (3.0–7.0)			
Skin or soft tissue infection	336	214 (64)	62 (19)	11.0 (7.0–15.0)	10–14 days	44	16
Other (not specified)	157	49 (31)	63 (19)	6.0 (3.0–10.0)			
Total	3390	1502 (44)	60 (19)	7.0 (4.0–11.0)			
Excluded****	232						

^*^Proportions are only given for treatment courses with post-discharge treatment

^**^Recommended by Dutch guidelines (www.swabid.nl)

^***^ One additional day was allowed as each calendar day on which a dose was given was counted as a full day of treatment

^****^ Indications that were rarely selected, i.e. in less than 20 treatment courses, or that would require > 21 days of treatment according to our national guidelines were excluded. These included the following indications: Bone and Joint infection, S. aureus bacteraemia, Endovascular infection, Eye infection, Febrile neutropenia, Fungal infection, Mediastinitis, UTI–cyst infection and chronic prostatatitis

*SD* Standard deviation, *UTI* Urinary tract infection, *UTI-CAD* Catheter-associated urinary tract infection, *RTI* Respiratory tract infection, *CAP-m* Community-acquired pneumonia mild-to-moderate severe, *CAP-s* Community-acquired pneumonia severe, *HAP* Hospital-acquired pneumonia, *COPD* Chronic obstructive pulmonary disease, *CNS* Central nervous system, *CVL* Central venous line

### Guideline adherence of total duration of antibiotic therapy

The guideline recommended total duration of therapy per indication and the adherence rate per indication are also presented in Table 2. Records were excluded from adherence evaluation in case accepted guideline recommendations were not available or in case the indication “other” was selected. Guideline adherence regarding duration of therapy ranged from 26 to 75%. The proportion of non-compliance because of excessive treatment duration varied from 10 to 50%. Prescriptions for RTI were most often considered to be too long, in particular aspiration pneumonia and mild-to-moderate severe CAP.

## Discussion

This study shows that it is feasible to reliably extract from the EMR the total duration of antibiotic therapy, broken down per indication and including post-discharge treatment, enabling an automated assessment of the appropriateness of antibiotic treatment duration. General inspection, and if indicated, optimization of the dataset (e.g., excluding erroneous prescriptions) is necessary to enable the use of data for further analysis. The total duration of therapy was accurately extracted from the EMR in 96% of infections, but the registered indication did not match the indication documented in the patient records in 17% of cases, which was mainly due to inaccurate selection of the mandatory indication in the EMR. Only 50% of these indication errors affected the evaluation of appropriateness of LOT, but local validation of the datasets is therefore necessary and the error-rate should be considered when data is used for quality measurement purposes. With regard to our secondary study aim, guideline non-adherence due to excessive treatment duration varied from 10 to 50% per indication, showing possibilities for quality optimization.

Including therapy after hospital discharge in the treatment duration is necessary, as a considerable amount of excess antibiotic use occurs after discharge, which was previously shown for CAP [2]. In our study, especially in ENT infection, complicated UTI and SSTI a high proportion (64%-83%) of antibiotic treatment courses was continued post-discharge, which made out 62–63% of total treatment duration. This emphasizes the need to include post-discharge prescriptions in the assessment of total treatment duration.

In previous studies the total duration of therapy linked to indication was either assessed by manual chart review [7, 8, 13] or by linking the antibiotic therapy to the ICD code [5, 6]. We used an electronic data extraction method, that can be used by all hospitals using EPIC software and that enables to assess a large amount of data more efficiently and more specifically. Determining the inclusion criteria and definitions, for example considering the last registered indication as the definitive treatment indication, and validation of the extracted data require time. However, as opposed to manual data assessment, the majority of time and expertise needs to be invested once, at the start of the project. Thereafter, surveillance can be performed automated as the syntax can be reused. A prerequisite is that the hospital EMR requires indication registration for each prescribed antibiotic. Although we did not show this in our study, the guideline-adherence of treatment duration and further specifications can be presented per hospital department, enabling the fine tuning of the targets for improvement.

Our inclusion criteria ensured a reliable dataset for the most common infections. We were able to confirm that by applying our inclusion criteria, the total duration of therapy was accurately extracted for almost all indications, with prescriptions in the OPAT setting being the most important exception. The error-rate of 4.5% of the electronically extracted LOT was far lower than the 11.5% discrepancy Dyer et al. found [6]. As electronic prescriptions are becoming the norm, we expect error-rates due to written prescriptions to drop further in the future. Unfortunately, 17% of prescriptions were linked to an inaccurately selected indication, of which half affected the assessment of guideline-adherent LOT. In our previous study we found similar proportions of inaccurately selected indications in the hospitals where the mandatory indication registration was recently introduced [10]. Saini et al., recently showed that the most common barriers for accurate documentation of the prescribing indication are uncertainty in diagnosis, time, logistical challenges and alert fatigue [14].

Based on the Capability-Opportunity-Motivation Behaviour model, designed by Michie and colleagues, behaviour can only occur when an individual has the capability, opportunity and motivation (including habitual process) to perform the behaviour [15]. The physicians of the participating centres did not receive any information yet about why the mandatory indication registration was implemented in the prescribing software and did not receive any feedback of their prescribing results, which may have caused lack of motivation. Furthermore, data was extracted one year after implementation of the mandatory indication registration. In our previous study we already saw that habituation decreases the error rate [10]. We therefore believe that the error rate can be decreased when information, education and feedback are given. Feedback can be given for example by providing benchmark results with other departments (locally) or other hospitals.

Finally, we showed the opportunities of using mandatory indication registration and assessment of the appropriateness of treatment duration to identify clear targets for ASP. The treatment duration for mild-to-moderate severe CAP, for example, was shown to be too long in 40% of patients, considering the guideline recommendation of five days. Each day of antibiotic therapy is associated with 4% increased odds of experiencing an adverse event [4]. For example, three days of ß-lactam therapy for CAP patients was shown to be non-inferior to eight days of therapy [16], while seven days of therapy instead of three is associated with a 1.19-fold increase in experiencing an adverse drug event [4]. This emphasizes the necessity for ASP to monitor treatment duration.

## Limitations

Due to our inclusion criteria we did not evaluate all antibiotic courses prescribed during hospital admission. We focused on patients that were initially empirically treated to enable reliable data extraction, as this method was already used and validated in our previous study [10]. In addition, we disregarded difficult-to-treat infections and nosocomial infections, as the local guidelines usually do not apply to these infections. Nevertheless, univocal local guideline recommendations were available for 51% (1745/3390) of the included treatment courses, in which guideline compliance could be assessed. We do believe that the records that were included gave a good representation of antibiotic use for the most common infections. Furthermore, we only evaluated antibiotic courses that were electronically prescribed. Written prescriptions, pre-admission prescriptions or antibiotics that were prescribed after transfer to other hospitals were therefore missed. When assessing the guideline adherence, this should be taken into account. The final point of consideration is the accuracy of the indication selection by prescribers. As human errors are inevitable and the error rate may fluctuate over time, the accuracy of the dataset should be checked regularly. This requires manual chart review, but that can be limited to a relatively small sample of patients, for example 10% of the extracted data.

## Conclusion

With this study we demonstrated that implementing mandatory indication registration in the EMR enables a reliable and efficient method for systemic registration of two core-elements of ASP: the guideline adherence of the total duration of antibiotic therapy and the guideline adherence of empiric antibiotic choice, the latter we showed in our previous study [10]. It has the potential to become a valuable aid for ASP, as it reduces the amount of manual data collection and has the ability to provide clear targets for local ASP. Datasets can be updated in a semi-automated fashion as the syntax can be reused whenever needed, enabling the regular surveillance of the extent and appropriateness of antibiotic use and the effect of ASP interventions. Regularly data validation, however, is necessary to assure accuracy of the extracted data. Furthermore, additional efforts (e.g. information, education and feedback) are important to increase the accuracy of the indication selection. The next steps are to implement mandatory indication registration in the outpatient setting, add microbiological results to the prescribing software for quality assessment of targeted therapy, and to utilize indicationselected at time of order entry for targeted real-time interventions, for instance using pre-defined order sets for specific indications in which the indication and duration are recommended.

## Supplementary Information

**Additional file 1.** Electronic medication registration prescription format.

## Data Availability

We will share aggregated, anonymized data, which have been used for this publication, upon reasonable request. We will share the (pseudo-)code/syntax used for this article upon reasonable request.
