# Supplementary material for: Detecting inappropriate total duration of antimicrobial therapy using semi-automated surveillance
Source: Antimicrob Resist Infect Control. 2022 Aug 29;11:110. doi: 10.1186/s13756-022-01147-2 (PMC9426230; doi:10.1186/s13756-022-01147-2)
Supplement: Supplementary file 1 — Additional file 1. Electronic medication registration prescription format. [file 13756_2022_1147_MOESM1_ESM.docx]

## **Supplemental material**

**Table 1. Electronic medication registration prescription format**

| **1. Select antimicrobial agent** | |
| --- | --- |
| **2. Select indication** | □ Empirical therapy □ Targeted therapy □ Prophylaxis □ IV-oral switch |
| **3. Select focus of infection** | □ Bones and joints □ Central nervous system □ Gastrointestinal □ Yeast/mycosis  □ Gynaecological infection □ Skin and soft tissue □ Intra-abdominal infection □ Ear-nose- and throat infection □ Febrile neutropenia □ CVL infection □ Respiratory tract infection □ Mediastinum □ Eye □ Staphylococcus aureus bacteraemia □ Urinary tract infection  □ Unknown/sepsis of unknown cause □ Other |
| **4. Select specified indication** | |
| ***In case of Respiratory tract infection*** | **□** Bronchitis- exacerbation of COPD □ Community acquired pneumonia – severe  □ Community acquired pneumonia mild to moderate severe □ Aspiration pneumonia  □ Hospital acquired pneumonia □ Lung abscess/pleural empyema □ Other |
| ***In case of Urinary tract infection*** | □ Cystitis □ Prostatitis, chronic □ Pyelonephritis □ Catheter-associated urinary tract infection □ Polycystic kidney urinary tract infection □ Urosepsis  □ Urinary tract infection in renal transplant |
| ***In case of Febrile neutropenia*** | □ Low-risk neutropenia □ High-risk neutropenia □ Not applicable |
